# Glenohumeral Pathologies following Primary Anterior Traumatic Shoulder Dislocation—Comparison of Magnetic Resonance Arthrography and Arthroscopy

**DOI:** 10.3390/jcm12216707

**Published:** 2023-10-24

**Authors:** Oliver Holub, Jakob E. Schanda, Sandra Boesmueller, Marion Tödtling, Alexander Talaska, Rudolf M. Kinsky, Rainer Mittermayr, Christian Fialka

**Affiliations:** 1 AUVA Trauma Center Vienna-Meidling, Department for Trauma Surgery, 1120 Vienna, Austria; jakob.schanda@auva.at (J.E.S.); sandra.boesmueller@auva.at (S.B.); marion.toedtling@auva.at (M.T.); rainer.mittermayr@auva.at (R.M.); christian.fialka@auva.at (C.F.); 2 Ludwig Boltzmann Institute for Traumatology, The Research Center in Cooperation with AUVA, 1200 Vienna, Austria; 3 Austrian Cluster for Tissue Regeneration, 1200 Vienna, Austria; 4 AUVA Trauma Center Vienna-Meidling, Department for Radiology, 1120 Vienna, Austria; alexander.talaska@auva.at; 5 Sanatorium Hera, 1090 Vienna, Austria; maximilian.kinsky@sanatoriumhera.at; 6 Medical Faculty, Sigmund Freud University Vienna, 1020 Vienna, Austria

**Keywords:** traumatic anterior shoulder dislocation, Hill–Sachs lesion, Bankart lesion, superior labrum from anterior to posterior lesion, magnetic resonance arthrography, arthroscopy

## Abstract

Background: We assessed intraarticular injuries in patients after primary anterior traumatic shoulder dislocation by comparing magnetic resonance arthrography (MRA) results with concomitant arthroscopic findings. Methods: All patients with primary traumatic shoulder dislocation who underwent arthroscopic surgery between 2001 and 2020 with preoperative MRA were included in this study. MRA was retrospectively compared with arthroscopic findings. Postoperative shoulder function was prospectively assessed using the Disabilities of Arm, Shoulder and Hand score (quick DASH), the Oxford Shoulder Score (OSS), the Subjective Shoulder Value (SSV), as well as the rate of return to sports. Results: A total of 74 patients were included in this study. A Hill–Sachs lesion was consistently found in the corresponding shoulders on MRA and arthroscopy in 35 cases (*p* = 0.007), a Bankart lesion in 37 shoulders (*p* = 0.004), and a superior labrum from anterior to posterior (SLAP) lesion in 55 cases (*p* = 0.581). Of all cases, 32 patients were available for a clinical and functional follow-up evaluation. A positive correlation was found between the level of sport practiced and the Oxford Shoulder Score (redislocation subset) (*p* = 0.032) and between the age at the time of surgery and the follow-up SSV (*p* = 0.036). Conversely, a negative correlation was observed between the age at the time of surgery and the Oxford Instability Score (redislocation subset) (*p* = 0.038). Conclusions: The results of this study show a good correlation between MRA and arthroscopy. Therefore, MRA is a valid tool for the detection of soft tissue pathologies after primary anterior traumatic shoulder dislocation and can aid in presurgical planning.

## 1. Introduction

With an overall incidence ranging from 24 to 56 per 100.000 persons/year, the glenohumeral joint has the highest dislocation rate of all joints [1,2]. Antero-inferior shoulder dislocation is the most common, occurring in 95% of all glenohumeral instabilities [3]. In the case of traumatic anterior shoulder dislocations, bony defects of the glenoid are reported in 5% to 56% of cases [4,5], whereas bony defects of the humerus, including Hill–Sachs lesions, are documented in 71% [6,7]. Depending on the recurrence rate of shoulder dislocations, tears or detachment of the labrum are reported in up to 100% [8]. In particular, superior labrum from anterior to posterior (SLAP) lesions are seen in 16% to 33% of cases [8]. Injuries of the postero-superior rotator cuff (RC) following traumatic shoulder dislocations occur in around 30% of patients older than 40 years of age, increasing up to 80% in patients over 60 years of age [9,10,11,12,13,14]. On the other hand, lesions of the subscapularis are observed in 21% of patients after traumatic anterior shoulder dislocations [15]. Magnetic resonance imaging (MRI) is considered the gold standard for detecting soft tissue injuries of the shoulder joint [16]. However, for the investigation of chronic labral lesions as well as of SLAP injuries, magnetic resonance arthrography (MRA) remains superior to MRI. MRA imaging maximizes anatomical resolution and enhances soft tissue contours by the additional application of an intraarticular contrast agent [17,18]. Still, there are inconclusive reports of the sensitivity and specificity of MRI and MRA concerning soft tissue injuries after traumatic anterior shoulder dislocation compared to arthroscopic findings [17]. This could pose a challenge in presurgical planning. The primary objective of this study was to investigate soft tissue lesions following traumatic first-time anterior shoulder dislocation, comparing MRA with arthroscopic findings. The secondary objective was to examine the association between intraarticular pathologies and redislocation rate after arthroscopic Bankart repair.

## 2. Materials and Methods

### 2.1. Study Population 

**Figure 1:**
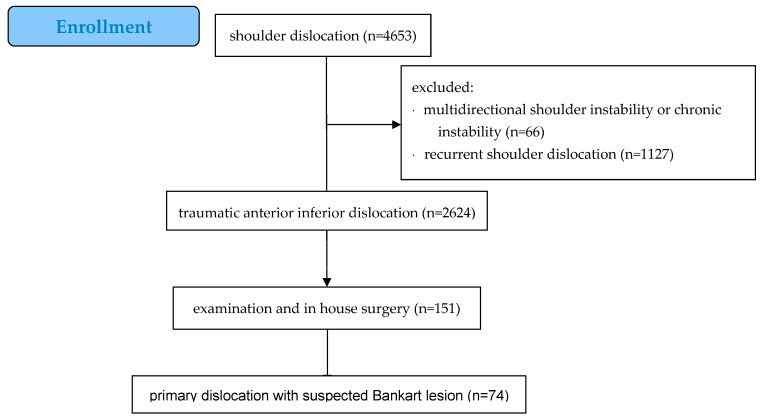
Enrollment process.

A total of 2624 patients between 16 and 60 years of age had primary anterior traumatic shoulder dislocation, of whom 151 underwent arthroscopic surgery. From this collective, 74 patients who had an arthroscopic Bankart repair between January 2001 and September 2020 were included in this study [Figure 1]. Exclusion criteria were posterior or recurrent shoulder dislocation, chronic or multidirectional shoulder instability, bony Bankart fractures, epilepsy, and alcohol/drug abuse. 

After informed and signed consent, the various scores (Subjective Shoulder Value—SSV, quick DASH, Oxford Shoulder Instability Score) were acquired in person, by phone, or by email. 

### 2.2. Radiological Assessment 

A single radiologist with extensive experience in musculoskeletal radiology retrospectively reviewed and analyzed all MRA scans (AT) in a blinded fashion. All studies were performed on two different MRI devices—Siemens^®^ Avanto 1.5T with a dedicated shoulder coil (Siemens^®^, Munich, Germany) for examinations after 2011 and Siemens^®^ 0.9T (Siemens^®^, Munich, Germany) for examinations before 2011. Intraarticular contrast agent was applied by a shoulder surgeon via a dorsal approach. Up to 15 mL of Magnevist^®^ (Bayer^®^, Leverkusen, Germany) was instilled into the glenohumeral joint under aseptic conditions. MRA imaging was performed with the affected shoulder in a prone position, with the affected arm in adduction and neutral rotation. The MRA protocol included axial, paracoronal, and parasagittal PD BLADE FS and coronal T1 TSE. Slice thickness was set at 2 mm after 2011 and 3 mm before 2011. Results of the MRA scans were compared to the surgical reports and intraoperative photo documentation by a single examiner (O.H.). If any information was missing, intraoperative images were taken for examination. 

### 2.3. Arthroscopic Bankart Repair 

**Figure 2:**
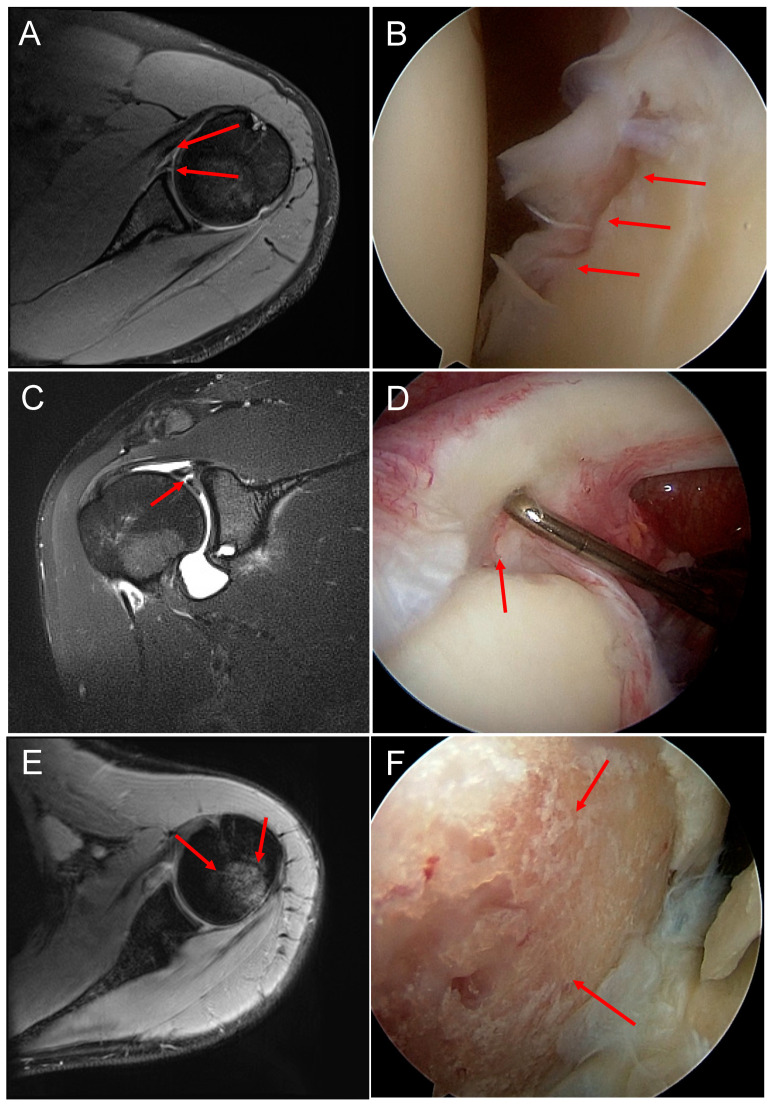
(**A**) MR image of a Bankart lesion (red arrows). (**B**) Corresponding arthroscopic image of a Bankart lesion. (**C**) MR image of a SLAP lesion. (**D**) Corresponding intraoperative image of a SLAP lesion. (**E**) MR image of a Hill–Sachs lesion. (**F**) Arthroscopic image of a Hill–Sachs lesion.

Arthroscopic Bankart repair was performed in the beach-chair position under general anesthesia with an interscalene blockade in all cases. The affected arm was secured in a hydraulic arm brace (Trimano™, Arthrex, Naples, FL, USA). After a standardized arthroscopic visualization of the glenohumeral joint and documentation of all pathological findings, the detached ventral labrum was first mobilized using an arthroscopic rasp. The glenoid was roughened using a shaver to enhance healing in the ruptured labrum. The capsule–labrum complex was armed distally and shifted cranially on the ventral glenoid border using a SutureLasso™ (Arthrex, Naples, FL, USA). Depending on the size of the Bankart lesion, the capsule–labrum complex was secured on the ventral glenoid border using two to three 3.5 mm PEEK PushLock™ anchors (Arthrex, Naples, FL, USA). The stability of the ventral capsule–labrum complex was tested using a probe. The postoperative plan for most patients consisted of wearing a shoulder sling for 4 weeks constantly. The sling was only to be removed for passive movement of the shoulder and range-of-motion exercises of the elbow. No external rotation of the shoulder was allowed for 6 weeks. Physiotherapy started after the shoulder sling was removed [Figure 2].

### 2.4. Clinical and Functional Evaluation 

All clinical and functional assessments were performed before and at least one year after surgery by a single examiner (O.H.). Postoperative patient-reported outcome measures (PROM) involved the quick Disabilities of the Arm, Shoulder, and Hand score (qDASH) (0: best; 100: worst) [19], the Oxford Shoulder Score (12: best; 60: worst) [20], and the Subjective Shoulder Value (SSV) (percentage of a 100% normal shoulder) [21]. 

### 2.5. Statistical Analysis 

For the detection of similar findings between MRA and arthroscopic results, a contingency table was first created for each individual parameter. Kappa was calculated with the following interpretation: <0, no agreement; 0.00 to 0.20, slight; 0.21 to 0.40, fair; 0.41 to 0.60, moderate; 0.61 to 0.80, substantial; 0.81 to 1.00, almost perfect agreement [22]. To evaluate the statistical significance between MRA and arthroscopic findings, a Wilcoxon matched pairs signed-rank test was performed, followed by a nonparametric distribution (D’Agostino and Pearson test). The difference in the assessment (injury yes/no) was determined pairwise between MRA and arthroscopy. For postoperative clinical and functional follow-up, correlations were assessed with the nonparametric Spearmen correlation test. Statistical significance was set at the conventional *p*-value of <0.05 (two-sided). All statistical analyses were carried out using GraphPad Prism (version 9.3.1/350).

## 3. Results

### 3.1. Patient Demographics 

Between January 2001 and September 2020, a total of 74 patients (female n = 12, male n = 62), with a mean age of 30.4 ± 11.8 years, suffered from primary traumatic anterior shoulder dislocation and underwent arthroscopic Bankart repair. The mean time between MRA and arthroscopic Bankart repair was 40 ± 41 days. The mean body mass index (BMI) was 25.1 ± 3.8 and the mean height was 180 ± 8 cm.

### 3.2. Comparison Magnetic Resonance Arthrography and Arthroscopy 

An analysis of the specificity of the initial MRI findings compared with arthroscopic lesions described later revealed a total of 74 concordant pathologies, mainly typical dislocation-associated changes such as the Hill–Sachs defect and Bankart lesion. However, a Hill–Sachs defect was diagnosed 30 times more in the MRA than could be found in the arthroscopic report (*p* < 0.0001). 

Conversely, no Bankart lesion was detected in 18 MRA cases, but turned out to be present at surgery (*p* = 0.0106). Thus, both of the above parameters showed poor agreement (Cohen’s kappa 0.029 and 0.070, respectively). Glenoid cartilage damage was almost inherently associated with a dislocation but was described in only three MRA cases. Based on surgery as the most reliable diagnostic method, 40 such cartilage defects were described in the operative report, and thus false positives were reported three times. More important are the 25 false negative findings in the MRA (*p* < 0.0001), although the respective cartilage defect only affected the immediate vicinity of the sheared labrum and was addressed surgically by labral reconstruction alone [Table 1].

In addition to the primary dislocation-associated lesions (Hill–Sachs, Bankart, cartilaginous glenoid defect), concomitant pathologies were numerous, with a total of 46 occurrences (Table 2). With decreasing frequency, SLAP (n = 14) and supraspinatus tendon lesion (n = 12), cartilaginous defect at the humeral head (n = 9), and proximal long biceps tendon lesion (n = 7) were found arthroscopically. In MRA, there were only two matches, 18 false positives, and 40 false negatives, compared with surgery. Just the SLAP lesion, with 11 false negative findings in MRA, again showed only a slight agreement (kappa 0.048). A direct pairwise comparison between MRA and surgery showed no statistically significant difference (*p* = 0.4807). Similarly, the supraspinatus tendon lesion had a slight Cohen’s kappa of 0.040 but no statistically significant difference when comparing the MRA report to operative findings. In addition to the biceps tendon anchor (SLAP), a lesion of the proximal long biceps tendon (LHBT) was also relatively common, with n = 7. There was no single injury of the LHBT described on MR tomography, and this was also significant, with *p* = 0.0312 in the pairwise statistical comparison of the findings (MRA vs. OR) [Table 2].

### 3.3. Measures during Surgery 

A Bankart repair was performed in all but five cases. Two arthroscopic operations were converted to open repairs: one for a large glenoid bone defect which was then addressed with screw osteosynthesis, and one for a complicated reconstruction of the supraspinatus tendon combined with tenodesis of the LHBT. Surgical procedures for bony pathologies were performed five times: three resections for failure to reconstruct glenoid fragment/bony Bankart, one Hill–Sachs defect with remplissage, and one glenoid screw fixation. Surgical intervention was performed in all but four cases (n = 70), regardless of whether the lesion was Bankart, SLAP, or rotator cuff (n = 69, n = 6, and n = 7, respectively). The number of PEEK PushLock (TM) anchors used for Bankart repair ranged from one (n = 3) to six (n = 1; four anchors for Bankart repair, two additional anchors for SLAP repair). Most cases were treated with two (n = 27) and three anchors (n = 34). 

### 3.4. Follow-Up Examinations 

A total of 32 patients consented (43% of the retrospective study) to follow-up (FU) after anterior shoulder dislocation and subsequent arthroscopic Bankart repair. The mean follow-up period for the two women and thirty men (6% vs. 94%, respectively) was 47.8 months (range: 7.5 to 149.9 months). The mean age of these patients at the time of surgery was 30.6 years (±2 SEM). Of those who have been followed up, the surgically detected lesions are depicted in [Table 3].

Mean postoperative qDASH was 7.2 ± 1.3, mean postoperative Oxford Shoulder Score was 41.7 ± 1.3, and mean postoperative SSV was 83.5 ± 2.8. Determining the correlation of the collected scores with the age of patients at time of surgery revealed a statistically significant positive correlation with SSV (*p* = 0.0428) and the redislocation subset of the Oxford Instability Score (*p* = 0.0410). No statistically significant relationship was found concerning the total number of lesions detected or the total number of anchors implanted. The same was true for the Bankart, SLAP, LHBT, and rotator cuff lesions and the number of anchors implanted, with no statistical correlation with the scores assessed. Contrary to the lack of correlation with cartilaginous lesions at the humeral head, cartilage defects at the glenoid showed a statistically positive correlation with SSV (*p* = 0.0008). The extent of sports activities was divided into recreational sports (n = 21), professional sports (n = 6), and no sports (n = 6). The most frequently performed sports activities after arthroscopy included swimming, cycling, running, and fitness training. Evaluating the correlation of sports activity with the scores, it appears that no sports activity was associated with an increased redislocation rate, according to the subset of the Oxford Instability Score (*p* = 0.0217).

## 4. Discussion

This study showed a good correlation between preoperative MRA and arthroscopic findings regarding soft tissue and bony lesions after traumatic primary shoulder dislocation. The study cohort showed comparable results regarding Bankart, SLAP, and Hill–Sachs lesions. Preoperative MRI remains the gold standard for detecting soft tissue lesions around the glenohumeral joint, but still has limitations in detecting chronic injury patterns such as anterior labroligamentous periosteal sleeve avulsion lesions (ALPSA). On the contrary, MRA is a valid tool for detecting soft tissue lesions, especially of the glenoidal labrum. New innovative planning techniques are being researched. Moldovan et al. developed a segmentation process of imagistic volumetric data in fractures. In the future, it may be possible to apply this technique to soft tissue injuries such as shoulder dislocation [23].

As reported by Genovese et al., MRA is useful to identify lesions affecting the superior portion of the shoulder, including location, morphology, extent, and associated injuries and lean anatomical variants, and to correlate these features with clinical symptoms [24]. MRA as a first-choice imaging modality has already been described by several authors [18,25,26,27,28]. Although MRA is a useful first-hand tool, as described above, there are still some statistical discrepancies between MRA and arthroscopy. 

Defects of the humeral bone, especially in the form of Hill–Sachs lesions, can be found in up to 71% of patients with a primary shoulder dislocation and can go up to 93% for recurring dislocations [4,5]. As for the detection of Hill–Sachs lesions, Vopat et al. in their 2021 systematic review observed a specificity range of 50–98% and a sensitivity range of 69–100% [29]. In our study, Hill–Sachs lesions were mostly found in MRA, which was expected. We could, however, show that a total of four Hill–Sachs lesions could only be diagnosed during surgery. This brings up the question if those four lesions were of recent nature or if the timeline between the accident and examination was too long. The fact that, in most cases, Hill–Sachs lesions cannot be improved surgically results in minimal consequences for the patients. 

Bankart lesions occur in a high number of traumatic shoulder dislocations, varying from 97% to 100% [30,31]. In 2017, Saqib et al. showed MRA to have a sensitivity of 60% for detecting Bankart lesions [32]. In our study, Bankart lesions were found using MRA in 61% of cases (n = 45). Regarding arthroscopic findings, a Bankart lesion was observed in 91% (n = 67). Interestingly, many Bankart lesions were solely seen during surgery, which could then be addressed accordingly. 

Referring to SLAP lesions, Saqib et al. showed MRA to have a high specificity. They found specificity to be higher than sensitivity in most lesions except labral tears [32]. In a study by Habermeyer et al., the authors found SLAP lesions in 16–33% of patients with posttraumatic shoulder injuries, depending on the frequency of shoulder dislocation [8]. This is in accordance with our findings of approximately 19% in our collective. In our present study, SLAP lesions could be detected surgically and with MRA in many cases.

In 2013, Lenza et al. did not show any statistical significance in comparing MRA to MRI or ultrasound in terms of detecting full-thickness rotator cuff tears [33]. However, in a recent meta-analysis from 2020, Liu et al. assessed the diagnostic value of MRA. The authors found a pooled sensitivity of 0.97 and specificity of 0.97 of MRA to detect any tear of the rotator cuff [25]. Rotator cuff injuries are common in patients with traumatic shoulder dislocation, as Itoi and Tabata described [9]. Numbers vary between 30% and 80% depending on the age of patients [9,11,12], with higher numbers in older patients. In our present study, a good correlation between MRA and arthroscopic findings of rotator cuff injuries could be found in 73–92% of patients (n = 54 for SSP to n = 68 for TM). The total number of rotator cuff injuries within our collective was relatively low, with 22% (n = 16). This could be explained by the young age of our patients.

It is of utmost importance for the surgeon to pay extra attention to pathologies with a high false negative rate in MRA, as seen in cartilage defects of the glenoid or Bankart lesions (34%, n = 25; 24%, n = 18).

Follow-up data show good patient satisfaction after surgical repair. The number of patients who suffer from redislocation after arthroscopic Bankart repair has a high variance in current literature and ranges from 0 to 19% [34,35]. Redislocation rates were low in our collective, comparable with the results of Eren et al., who found a redislocation rate of 7.4% five years following arthroscopic Bankart repair [36]. Patients who suffered from redislocation were under the age of 21 years. This is supported by the literature, which shows age as one risk factor resulting in instability after surgery [37]. Patients who practice sport regularly had a lower risk of shoulder redislocation. This shows that sports activities can have a protective effect on redislocation rates. A relative new field in the treatment of sports injuries is stem cell therapy. Clinical outcome results are promising. Extensive research is currently underway and will continue in the future [38]. Vermeulen et al. [39] found that using less than three anchors in surgery to address labral tears was associated with a higher incidence of redislocation. Although most of our patients received two or three anchors, the number of anchors used in surgery did not play a significant role in any of the scores.

### Limitations

The main limitation of this study was the total number of patients included in this study; this is primarily due to our very selective patient cohort (primary traumatic shoulder dislocation, MRA, monocentric study). The fact that we could not reach all patients for the follow-up questionnaire resulted in a relatively high loss to follow-up rate. Another drawback was the lack of a standardized postsurgery regimen, i.e., physiotherapy.

## 5. Conclusions

The results of this study show that due to the good correlation between MRA and intraoperative findings, MRA is a valid tool to detect pathologies and can therefore be used in presurgical planning. Consequently, we suggest an early examination via MRA after trauma. There is no strict need for a contrast medium, because early hemarthrosis can act as a natural contrast medium. It provides pertinent preoperative information about the exact localization of tears and involvement of the bicep tendon. Additionally, the patient can profit from better presurgical informed consent. Pathologies that cannot be detected as effectively, e.g., cartilage defect of the humoral head or glenoid, should be paid extra attention during surgery.

## Figures and Tables

**Table 1:** Results of comparison between operation (OR) and magnetic resonance arthrography (MRA).

Pathology	Total Number of Pathologies, OR	Total Number of Pathologies, MRA	Match in both OR and MRA with Pathology Present	Match in both OR and MRA with Pathology Absent	False Positive (Pathology Detected in MRA, Absent in OR)	False Negative (Pathology Detected in OR, Absent in MRA)	Kappa ^1^	*p*-Value ^2^
Hill–Sachs lesion	36	60	31	5	30	4	0.029	**<0.001**
Bankart lesion	67	45	41	1	5	18	0.070	**0.011**
ALPSA	0	1	0	67	1	0	n.a.	>0.999
SLAP	14	8	1	49	7	11	0.048	0.481
HAGL	0	1	0	67	1	0	n.a.	>0.999
Greater tuberosity fracture	0	5	0	64	4	0	n.a.	0.125
Cartilage lesion humerus	9	1	0	58	1	9	n.a.	**0.022**
Cartilage lesion glenoid	29	3	0	40	3	25	n.a.	**<0.001**
Supraspinatus lesion	12	4	1	53	3	11	0.040	0.057
Infraspinatus lesion	1	1	0	66	1	1	n.a.	>0.999
Subscapularis lesion	3	0	0	66	0	2	n.a.	0.500
Teres minor lesion	0	0	0	68	0	0	n.a.	n.a.
Long head of the biceps tendon lesion	7	0	0	62	0	6	n.a.	**0.031**

Significant *p*-values are marked in bold. n.a., not applicable. ^1^ Kappa indicates degree of match between magnetic resonance arthrography and arthroscopic findings. ^2^ *p*-values indicate difference between magnetic resonance arthrography and arthroscopic findings using Wilcoxon matched pairs signed-rank test. ALPSA, anterior labroligamentous periosteal sleeve avulsion; HAGL, humeral avulsion of the glenohumeral ligament; SLAP, superior labrum anterior–posterior.

**Table 2:** Intraoperative pathologies in numbers and percentage of total shoulders. Abbreviations as in Table 1.

Pathology	n	%
Hill–Sachs	36	50%
Bankart lesion	67	91%
ALPSA	0	0%
SLAP	14	18%
HAGL	0	0%
GT fracture	0	0%
CartDefect H	9	13%
CartDefect G	29	37%
SSP lesion	12	18%
ISP lesion	1	1%
SSC lesion	3	3%
TM lesion	0	0%
LHBT	7	9%

**Table 3:** Intraarticular pathologies found during surgical repair in FU cohort (n = 32). Abbreviations as in Table 1.

Pathology	n	%
Hill–Sachs	15	47%
Bankart lesion	29	91%
ALPSA	0	0%
SLAP	10	31%
HAGL	0	0%
GT fracture	0	0%
CartDefect H	5	16%
CartDefect G	12	38%
SSP lesion	2	6%
ISP lesion	1	3%
SSC lesion	2	6%
TM lesion	0	0%
LHBT	3	9%
